# Serum Complement Factor H: A Marker for Progression and Outcome Prediction Towards Immunotherapy in Cutaneous Squamous Cell Carcinoma

**DOI:** 10.3390/cancers17132162

**Published:** 2025-06-26

**Authors:** Glenn Geidel, Laura Adam, Sabrina Bänsch, Nathan Fekade, Benjamin Deitert, Alessandra Rünger, Julian Kött, Tim Zell, Isabel Heidrich, Daniel J. Smit, Klaus Pantel, Stefan W. Schneider, Christoffer Gebhardt

**Affiliations:** 1 Department of Dermatology and Venereology, University Medical Center Hamburg-Eppendorf, 20246 Hamburg, Germany; g.geidel@uke.de (G.G.);; 2 Fleur Hiege Center for Skin Cancer Research, University Medical Center Hamburg-Eppendorf, 20246 Hamburg, Germany; 3 Institute of Tumor Biology, University Medical Center Hamburg-Eppendorf, 20246 Hamburg, Germany

**Keywords:** complement factor H, liquid biopsy, immunotherapy, cemiplimab, cutaneous squamous cell carcinoma

## Abstract

**Background/Objectives:** Tumor-immune system interactions shape the progression of cutaneous squamous cell carcinoma (cSCC). Serum biomarkers for risk stratification remain limited. Complement factor H (CFH) regulates the alternative complement pathway. It has been linked to immunosuppression and cSCC development in tissue-based studies. We investigated whether serum CFH is associated with tumor aggressiveness and may help predict immunotherapy outcomes in advanced cSCC. **Methods:** In this retrospective, single-center study, pre-treatment serum CFH levels were measured in 104 cSCC patients (62 high-risk and 42 advanced) using ELISA. Associations with clinical characteristics, disease stage, and response to cemiplimab were analyzed. Subgroup comparisons considered immune status and inflammatory comorbidities. **Results:** Advanced cSCC patients had significantly higher CFH levels than high-risk patients (OR 0.13, *p* = 0.026), independent of tumor diameter or invasion depth. Among advanced cSCC cases, lower baseline CFH was associated with more prolonged progression-free survival (median 19.8 vs. 3.07 months, *p* = 0.029; HR 0.29, *p* = 0.014), independent of covariates including immunosuppression. CFH levels during therapy did not predict treatment response. ROC analysis showed moderate discriminatory ability with CFH alone (AUC 0.625), which improved when combined with clinical variables in a multivariable risk model (AUC 0.767). **Conclusions:** Serum CFH is an independent predictor of cemiplimab response and reflects biological aggressiveness in cSCC beyond conventional high-risk features. These findings support the use of CFH in clinical risk models and warrant external validation in multicenter cohorts.

## Simple Summary

Cutaneous squamous cell carcinoma is a common form of skin cancer that is usually curable with surgery. However, some tumors grow aggressively, spread, or become unresectable. In these cases, systemic therapies like immunotherapy may be required. Predicting which tumors will behave aggressively or respond to treatment remains challenging. In this study, we measured the blood levels of a protein called complement factor H in patients with different stages of cutaneous squamous cell carcinoma. We found that higher levels of complement factor H were associated with more advanced disease and poorer responses to immunotherapy. Our results suggest that complement factor H may serve as a simple blood-based marker to identify patients with aggressive cutaneous squamous cell carcinoma earlier. It could also aid in predicting how patients might respond to immunotherapy. These insights may help clinicians individualize surveillance and treatment strategies for this disease.

## 1. Introduction

Cutaneous squamous cell carcinoma (cSCC) is the second most common skin cancer worldwide, with a rising incidence driven by ageing populations and chronic ultraviolet (UV) exposure [1,2,3]. While early-stage cSCC typically has a favorable prognosis, a subset of tumors displays high-risk features that are associated with increased risk of recurrence or metastasis [4,5,6,7,8]. These include a large tumor diameter, deep invasion, poor differentiation, or perineural spread. However, high-risk cSCC (hi-cSCC) remains surgically resectable in most cases [9]. In contrast, advanced cSCC (adv-cSCC), defined by irresectability, nodal metastasis, or distant dissemination, requires an individualized approach, including immune checkpoint inhibition (ICI) as a first-line treatment option [10]. Accurate distinction between high-risk and advanced disease remains critical to guide surgical decision-making, surveillance intensity, and the timely escalation to systemic treatment [11,12,13].

Recent insights into tumor immunology have identified the complement system as a central modulator of cancer progression [14,15,16]. Complement factor H (CFH) is a key regulator of the alternative complement pathway. It was found to be upregulated in various malignancies [17,18,19]. Riihilä et al. reported an increased expression of CFH in cSCC tumors compared to normal skin [20]. These findings confirm that immunohistochemistry detects CFH at the tumor tissue level. More recently, Johnson et al. reported that sun exposure is associated with the early induction of CFH in tumor tissue [21]. The authors established a link between CFH and promoting a locally immunosuppressive immune environment. This immunosuppressive environment may promote tumor initiation and progression. The tissue-based findings align with recent reports in other cancer entities, where CFH has been identified as a novel innate immune checkpoint. Its function appears to facilitate immune evasion and promote tumor progression [22,23].

Despite growing evidence for the role of CFH in tumor immunity, its clinical utility as a systemic biomarker remains unexplored. While tissue-level CFH expression has been implicated in cSCC development, its systemic relevance and potential as a blood-based biomarker remain undefined. The present study addressed this gap by evaluating serum CFH concentrations in patients with cSCC stratified by clinical risk. Specifically, we assessed whether serum CFH levels could be a systemic biomarker of biologically aggressive disease phenotype associated with progression beyond surgical resectability. In addition, this analysis explored the potential of serum CFH to identify patients with advanced, systemically treated cSCC. Its ability to predict treatment response and survival outcomes under immune checkpoint inhibition was also investigated. We hypothesized that elevated serum CFH is associated with immune escape mechanisms and may be a surrogate marker of progression and treatment resistance. CFH could thus represent a clinically useful biomarker for patient stratification and risk-adapted therapeutic decision-making.

## 2. Materials and Methods

### 2.1. Patient Cohort

This single-center, retrospective observational study included 62 hi-cSCC and 42 adv-cSCC patients, for which pre-treatment blood samples were available. This cohort included consecutive patients presenting with high-risk or advanced cSCC to our center between October 2019 and February 2025 who consented to serum biobanking. Pre-treatment peripheral blood samples were collected and stored as part of the institutional biobank at the University Skin Cancer Center Hamburg, which routinely archives serum, plasma, and peripheral blood mononuclear cells (PBMCs) for research purposes. Given the observational design and the rarity of advanced cSCC (approximately 1000 cases annually in Germany), no a priori power calculation was feasible or performed. However, a post hoc power analysis was conducted to assess the adequacy of our sample size. Based on the observed hazard ratio of 0.29 for progression-free survival (PFS), our cohort of 42 adv-cSCC patients provided >80% power to detect such a difference at a two-sided alpha of 0.05 using a log-rank test. While retrospective power analyses cannot substitute for a prospective design, this supports the interpretability of our findings in the context of a rare disease and real-world clinical sampling.

The classification of hi-cSCC was based on national guideline criteria and was defined by the presence of at least one clinical or histopathological high-risk feature. Clinical high-risk features included tumor location at a high-risk site (lower lip or ear), horizontal diameter ≥ 2 cm, local recurrence, immunosuppression, or fixation to underlying tissue. Histopathological high-risk features included invasion depth > 5 mm, desmoplasia, perineural invasion, subcutaneous extension, or poor histological differentiation (G3) [24]. Adv-cSCC was defined as irresectable disease due to extensive local infiltration or aggressive growth that is not amenable to surgery or radiotherapy, or as metastatic disease with regional or distant spread, in compliance with national guidelines [24]. All patients in the advanced cSCC cohort received systemic treatment with cemiplimab. Treatment response was evaluated using clinical examination and radiographic imaging (MRI and CT). In addition to the pre-treatment (baseline) time point, samples were collected six and twelve weeks after initiation of cemiplimab in adv-cSCC patients.

Demographic data included age, Eastern Cooperative Oncology Group (ECOG) performance status, and comorbidities. The “inflammatory condition” category included diseases like rheumatoid arthritis, hepatitis, Crohn’s disease, or psoriasis vulgaris. The category “autoimmune disease” included diseases like collagenoses or diabetes mellitus type I. The category “cardiovascular disease” included diseases like myocardial infarction, cerebral insult, peripheral arterial disease, or coronary heart disease. The category “immunosuppression status” included immunosuppressive medication such as prednisolone or biologicals, patients after organ transplant, or diseases like chronic lymphocytic leukemia. The category “hematologic neoplasm” was confined to myeloproliferative neoplasms such as polycythemia vera or chronic lymphocytic leukemia.

### 2.2. Complement Factor H Assay

Complement factor H (CFH) levels were measured in serum using the Human Complement Factor H ELISA Kit (Abcam, Cambridge, UK). All assays were performed according to the manufacturer’s instructions. Each ELISA plate included a whole standard curve using serial dilutions of recombinant CFH (0–1000 pg/mL), which served as the built-in positive control for assay calibration. Negative controls were included as blank wells containing assay buffer only, as specified by the kit protocol. All patient serum samples were assayed in duplicate, and final concentrations were determined by averaging replicate values. The intra-assay coefficient of variation (CV) was consistently below 10%, indicating high technical reproducibility. CFH levels were measured before surgical resection in hi-cSCC patients and before the treatment with cemiplimab in advanced cSCC patients.

### 2.3. Statistical Analysis

Statistical analysis was performed using RStudio v2024.09.0 with R v4.4.2, employing the packages “broom”, “maxstat”, “pROC”, “ggplot2” and “survival”. Graph-Pad Prism software version 10.4.0 was used to create scatter and bar graphs. The normality of continuous variables, including serum CFH levels, was assessed using the Shapiro–Wilk test. Group comparisons involving baseline CFH levels and the number of clinical, histological, and total high-risk features were analyzed using the Mann–Whitney U test, as these variables did not meet normality assumptions (Shapiro–Wilk *p* < 0.05). For these comparisons, the Hodges–Lehmann estimator was reported as a non-parametric measure of the difference in medians and exact two-tailed *p*-values were calculated. Comparisons involving CFH levels at 6 and 12 weeks were performed using unpaired *t*-tests. Longitudinal changes in CFH levels were assessed using a two-way repeated-measures ANOVA. Statistical significance was defined as *p* < 0.05. Correlation was evaluated using Spearman’s rank correlation coefficient, given the non-normal distribution of baseline CFH. The receiver operating characteristic (ROC) analysis assessed the discriminative performance of CFH and clinical covariates, with area under the curve (AUC) values calculated using DeLong’s method. Progression-free survival (PFS) and overall survival (OS) were analyzed using the Kaplan–Meier method, with group differences assessed via the log-rank test. Multivariable survival analysis was conducted using Cox proportional hazards regression, with hazard ratios (HRs), 95% confidence intervals (CIs), and corresponding *p*-values reported. Logistic regression (univariable and multivariable) was used to evaluate associations between clinical variables and disease stage (hi-cSCC vs. adv-cSCC).

## 3. Results

### 3.1. Cohort Characteristics

One hundred and four patients were included, comprising 62 hi-cSCC and 42 adv-cSCC (Table 1). The cohorts were comparable for age at diagnosis, sex, and overall comorbidity burden, including cardiovascular disease. Most patients in both groups were male, with a median age of 78 years in the hi-cSCC group and 80 years in the adv-cSCC group. Clinical high-risk features such as tumor location at the ear, lip, or temple, diameter > 2 cm, and immunosuppression were similarly distributed. In contrast, local recurrence and tumor fixation to underlying tissue were more frequent in the adv-cSCC group (Table 1). Histopathological features, including increased infiltration depth, perineural invasion, and poor differentiation, were observed in both cohorts, while infiltration beyond the subcutis was more common in adv-cSCC. ECOG performance status was slightly worse in the adv-cSCC group (Table 1). These baseline differences prompted further analysis of systemic CFH levels concerning disease stage and progression.

### 3.2. Expression of CFH in hi-cSCC and adv-cSCC Patients

**Figure 1:**
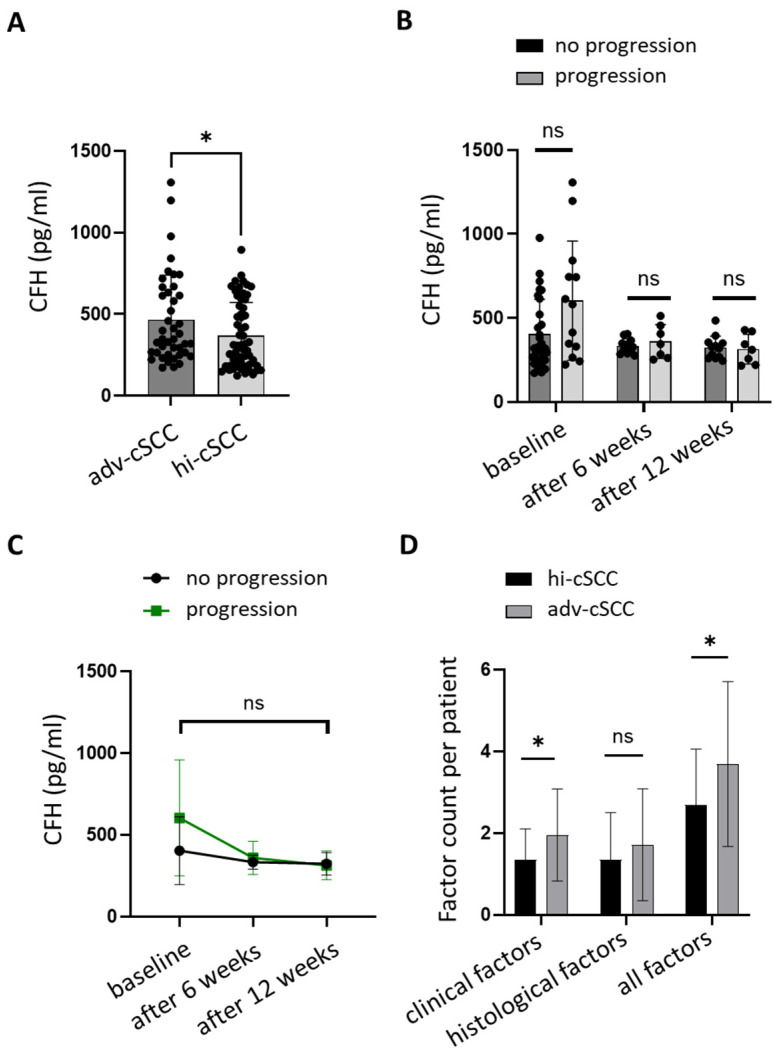
Serum levels of CFH in hi-cSCC and adv-CSCC and distribution of high-risk factors. (**A**) Pre-treatment complement factor H (CFH) levels in pg/mL per patient stratified by high-risk (hi-cSCC, median 302.7 pg/mL) and advanced cSCC (adv-cSCC, median 347.7 pg/mL); Mann–Whitney U test, *p* = 0.0305 (difference between medians: −44.97; Hodges–Lehmann estimator: −72.28); threshold for statistical significance was set at *p* ≤ 0.05. Group sizes: adv-cSCC n = 42, hi-cSCC n = 62. (**B**) CFH levels in the adv-cSCC group at baseline, week 6, and week 12, stratified by disease progression status (progression vs. no progression). At baseline *p* = 0.0816 (progression median = 580.6 pg/mL, no progression median = 319.6 pg/mL; median difference: +260.9 pg/mL; Hodges–Lehmann estimator: 124.1 pg/mL), at week 6 *p* = 0.9018 (progression median = 344.3 pg/mL, no progression median = 334.2 pg/mL; median difference: +10.1 pg/mL; Hodges–Lehmann estimator: 5.26 pg/mL), at week 12 *p* = 0.6504 (progression median = 310.7 pg/mL, no progression median = 323.0 pg/mL; median difference: −12.3 pg/mL; Hodges–Lehmann estimator: −13.99 pg/mL). All comparisons: Mann–Whitney U test, threshold for statistical significance was set at *p* ≤ 0.05. Group sizes: baseline (no progression n = 29, progression n = 13), after 6 weeks (no progression n = 12, progression n = 7), after 12 weeks (no progression n = 12, progression n = 7). (**C**) CFH levels across timepoints in adv-cSCC. Two-way ANOVA, significance level *p* ≤ 0.05: *p* = 0.148 (SE of difference 49.51, CI 95% −171.0 to 26.33). Group sizes: no progression n ≥ 12, progression n ≥ 7. (**D**) The count of clinical, histological, and total factors per patient is stratified by hi-cSCC and adv-cSCC. Clinical factors *p* = 0.0027 (adv-cSCC median = 2.0, hi-cSCC median = 1.0; median difference: +1.0; Hodges–Lehmann = 1.0), histological factors *p* = 0.205 (adv-cSCC median = 2.0, hi-cSCC median = 1.0; median difference: +1.0; Hodges–Lehmann = 0.0), all factors *p* = 0.0058 (adv-cSCC median = 4.0, hi-cSCC median = 2.5; median difference: +1.5; Hodges–Lehmann = 1.0); All comparisons: Mann–Whitney U test, threshold for statistical significance was set at *p* ≤ 0.05. Group sizes: adv-cSCC n = 42, hi-cSCC n = 62. SE: standard error, CI: confidence interval, ns: not significant. *, *p* < 0.01.

We first investigated whether serum CFH levels differed between high-risk and advanced cSCC patients, potentially reflecting disease severity. Baseline CFH concentrations were significantly higher in patients with advanced cSCC (median = 347.7 pg/mL) compared to high-risk cSCC (median = 302.7 pg/mL; *p* = 0.0305, Mann–Whitney U test; difference between medians: 44.97 pg/mL; Hodges–Lehmann estimator: –72.28 pg/mL) (Figure 1A). Among adv-cSCC patients, those with progressive disease (PD) exhibited higher baseline CFH levels than those achieving clinical benefit (CR, PR, or SD), although this difference did not reach statistical significance (median = 580.6 vs. 319.6 pg/mL; *p* = 0.0816; Figure 1B). CFH levels measured at six and twelve weeks after initiation of cemiplimab showed no significant differences between progressors and non-progressors (week 6: 344.3 vs. 334.2 pg/mL, *p* = 0.9018; week 12: 310.7 vs. 323.0 pg/mL, *p* = 0.6504; Figure 1B,C). We quantified the number of high-risk features per patient to assess whether established clinical risk factors could explain these CFH differences. Patients with adv-cSCC had significantly more total high-risk features than those with hi-cSCC (median = 4.0 vs. 2.5; *p* = 0.0058; Hodges–Lehmann = 1.0). This difference was driven by a higher number of clinical features (median = 2.0 vs. 1.0; *p* = 0.0027; Hodges–Lehmann = 1.0). In contrast, the number of histological features did not differ significantly between groups (median = 2.0 vs. 1.0; *p* = 0.2053; Hodges–Lehmann = 0.0) (Figure 1D).

### 3.3. CFH Is a Diagnostic Classifier for hi-cSCC and adv-cSCC Independent of Conventional High-Risk Features

**Figure 2:**
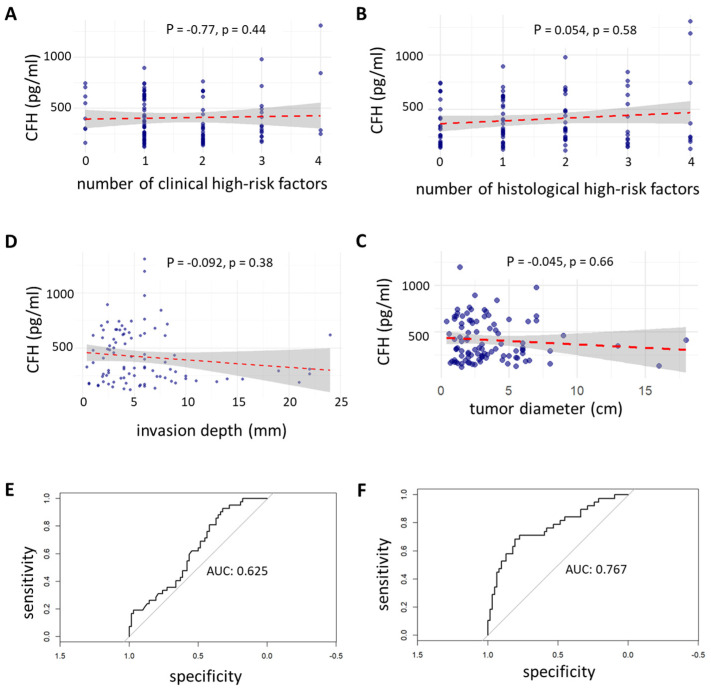
Correlation of CFH with high-risk features and ROC-based stratification. (**A**–**D**) Scatter plots using Spearman correlation (P = Rho) depicting serum CFH levels to (**A**) the number of clinical high-risk features, (**B**) histological high-risk features, (**C**) tumor diameter in cm, and (**D**) invasion depth in mm. Each plot includes a linear regression line (red dashes) with 95% confidence interval shading. (**E**) The receiver operating characteristic (ROC) curve shows the classification performance of serum CFH alone to distinguish high-risk from advanced cSCC. (**F**) ROC curve showing the classification performance of a multivariable model combining CFH, tumor diameter, number of clinical high-risk features, and immunosuppression status.

Next, we assessed whether serum CFH levels correlated with established clinical or pathological risk indicators. Spearman correlation analyses revealed no significant linear relationship between CFH levels and the number of clinical (P = −0.077, *p* = 0.44) or histological (P = 0.054, *p* = 0.58) high-risk features (Figure 2A,B).

Similarly, CFH levels did not significantly correlate with tumor diameter (P = −0.045, *p* = 0.66) or invasion depth (P = −0.092, *p* = 0.38) (Figure 2C,D), suggesting that CFH may capture a distinct aspect of tumor biology not reflected by conventional risk metrics. To assess the potential of CFH as a diagnostic classifier between hi-cSCC and adv-cSCC, we performed receiver operating characteristic (ROC) curve analysis. CFH alone yielded an area under the curve (AUC) of 0.625 (95% CI 0.52–0.73), indicating modest discriminatory ability (Figure 2E).

However, combining CFH with tumor diameter, clinical high-risk features, and immunosuppression status significantly improved classification performance, increasing the AUC to 0.767 (95% CI 0.67–0.87) (Figure 2F). Logistic regression analysis confirmed that low baseline CFH levels (<718.28 pg/mL) were independently associated with hi-cSCC rather than adv-cSCC (OR 0.13, 95% CI 0.02–0.70, *p* = 0.026) (Table 2). Additionally, a higher number of clinical high-risk factors independently predicted advanced disease status, whereas the total number of combined risk factors did not retain significance in multivariable analysis. Other covariates, including age, sex, and comorbidities, were not significantly associated with disease status. An ECOG performance status of 1 was significant in univariable analysis but not independent from other covariates in the multivariable model (Table 2).

### 3.4. Pre-Treatment CFH Independently Predicts PFS in adv-cSCC

**Figure 3:**
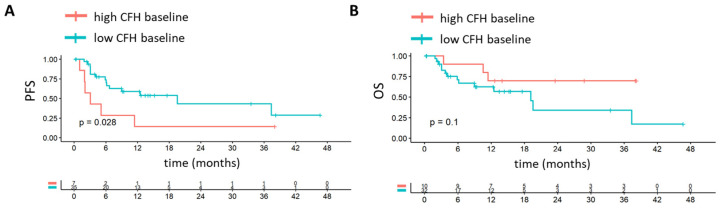
Kaplan–Meier analysis and distribution of pre-treatment CFH in adv-cSCC. (**A**,**B**) Optimized cutpoint (OCT) survival analysis for pre-treatment complement factor H (CFH) in advanced cutaneous squamous cell carcinoma (adv-cSCC) patients. (**A**) Progression-free survival (PFS) (median PFS: 19.53 [CI 95% 6.7 to not reached] vs. 3.07 months [CI 95% 1.97 to not reached]), OCT = 718.28 pg/mL; (**B**) overall survival (OS), OCT = 633.32 pg/mL.

Although baseline CFH levels were not statistically different between adv-cSCC patients with progression compared to those without progression, they showed a clear trend toward higher values in patients with disease progression. To further explore the clinical relevance of this observation, we performed Kaplan–Meier survival analysis stratified by baseline CFH levels. Patients with low CFH exhibited significantly longer PFS compared to those with high CFH levels (median PFS: 19.53 [CI 95% 6.7 to not reached] vs. 3.07 months [CI 95% 1.97 to not reached], *p* = 0.029) (Figure 3A). No significant association was observed between CFH levels and overall survival (OS) (Figure 3B).

We conducted uni- and multivariable Cox regression analysis to validate these findings. Low baseline CFH levels remained an independent predictor of prolonged PFS (HR 0.29, 95% CI 0.10–0.78, *p* = 0.014) (Table 3). CFH levels at six or twelve weeks after treatment initiation were not associated with progression outcomes, indicating that CFH serves best as a static baseline predictor. Interestingly, immunosuppression status emerged as a strong independent predictor of disease progression (HR 3.80, 95% CI 1.38–10.42, *p* = 0.009), while other clinical variables did not exhibit significant predictive value in multivariate models (Table 3).

## 4. Discussion

This study investigated serum CFH as a candidate biomarker for tumor aggressiveness and treatment outcomes in cSCC. We demonstrated that pre-treatment CFH levels were significantly higher in patients with advanced, systemically treated cSCC than in those with resectable high-risk disease. This difference was independent of tumor size or invasion depth, suggesting that CFH may reflect biologically aggressive tumor behavior not captured by conventional clinicopathological measures. Importantly, elevated CFH levels were independently associated with reduced progression-free survival (PFS) under immune checkpoint inhibition. This supports its role as a potential predictive marker for immunotherapy response.

These findings extend previous work on the role of CFH in oncologic immune evasion [22,25,26,27]. As a key regulator of the alternative complement pathway, CFH inhibits complement-mediated cytotoxicity by protecting cells from complement activation [28,29]. Prior studies, such as those by Riihilä et al. and Johnson et al., have shown elevated CFH expression in cSCC tissues and proposed a link between CFH and an immunosuppressive tumor microenvironment [17,20,21,30,31]. In the context of cSCC, prior studies have identified UV-induced CFH expression at the tissue level, contributing to an impaired innate immune system. Our data are the first to demonstrate that this immunomodulatory phenotype may also be mirrored systemically and serve as a clinically relevant serum biomarker.

From a translational perspective, serum CFH offers potential as a minimally invasive tool to refine existing risk stratification. Traditional indicators—such as tumor size, differentiation, and perineural invasion—often do not fully capture the likelihood of progression to unresectable or metastatic disease. In our ROC analysis, serum CFH alone showed moderate discriminative power (AUC 0.625), but performance improved markedly when combined with clinical variables (AUC 0.767). These findings support the integration of CFH into multivariate risk models and may help guide clinical decisions regarding the timing of systemic therapy or surveillance intensity. This is especially relevant in light of recent debates around adjuvant cemiplimab in high-risk cSCC, where predictive biomarkers remain an unmet need [13,32].

In light of this context, a particularly relevant subgroup is immunosuppressed patients characterized by impaired adaptive immunity [33,34,35]. In our cohort, immunosuppression was an independent predictor of disease progression in adv-cSCC under cemiplimab, consistent with its role as a clinical risk factor in cSCC [9]. In logistic regression analysis, immunosuppression did not significantly discriminate between high-risk and advanced cSCC. However, incorporating it into the multivariable ROC model alongside CFH and other clinical features improved the model’s discriminative performance. This suggests that while immunosuppression status alone may not discriminate disease status, it contributes additional risk stratification value when combined with biologically informative markers. Elevated CFH levels could help uncover a subgroup of immunosuppressed patients with particularly aggressive disease phenotypes, highlighting the need for risk-adapted management strategies in this vulnerable population.

Importantly, we demonstrated that CFH did not correlate with tumor burden indicators such as size or depth, distinguishing it from general markers like lactate dehydrogenase (LDH). This strengthens the hypothesis that CFH may capture tumor-intrinsic immune escape mechanisms rather than tumor mass alone [17,20,21]. If confirmed, this distinction would make CFH particularly valuable in identifying patients at biological risk of progression, even in the absence of overt clinical aggressiveness.

This study has several limitations. Its retrospective, single-center design may restrict generalizability, and causality cannot be established. CFH was measured primarily at baseline; although follow-up samples were included at weeks 6 and 12, longitudinal trends beyond this point were unavailable. Functional assays to confirm a mechanistic role of CFH in immune resistance were not part of this analysis and represent an important direction for future work.

Nevertheless, this study benefits from a well-defined, biobank-linked cohort of systemically treated cSCC patients with standardized clinical follow-up. Despite the rarity of advanced cSCC, we assembled one of the most extensive real-world series with serum profiling and outcome data under immunotherapy. All patients were managed under standardized institutional protocols, with rigorous data collection and outcome assessment, adding consistency and robustness to our findings.

## 5. Conclusions

This study identifies serum CFH as a promising biomarker of disease progression and immunotherapy response in cutaneous squamous cell carcinoma. Elevated pre-treatment CFH levels were independently associated with unresectable, biologically aggressive disease and shorter progression-free survival under cemiplimab, even after adjusting for established risk factors.

Our findings support the incorporation of CFH into clinical risk models to better stratify cSCC patients by biological risk and potential benefit from immunotherapy. Given its minimally invasive nature, serum CFH measurement may be well suited for integration into routine workflows and could inform decisions about surveillance and early systemic treatment.

Future research should focus on validating these results in multicenter cohorts, exploring the temporal dynamics of CFH during disease evolution, and clarifying its mechanistic role in immune evasion. These efforts will determine whether CFH could also serve as a therapeutic target or companion diagnostic in managing aggressive cSCC.

## Figures and Tables

**Table 1:** Patient characteristics. Clinical characteristics of high-risk cutaneous squamous cell carcinoma (cSCC) and advanced cSCC group. ECOG: Eastern Cooperative Oncology Group; CFH: complement factor H. NA: not applicable.

		High-Risk cSCC	Advanced cSCC
Total patients		62	42
Median age at diagnosis (years)		78 (55–90)	80 (55–98)
Sex			
	male	46 (74.1%)	33 (78.6%)
	female	16 (25.9%)	9 (21.4%)
Clinical high-risk factors			
	location: ear, lip, temple	15 (24.2%)	10 (23.8%)
	local recurrence	6 (9.6%)	15 (35.7%)
	diameter above 2 cm	43 (69.3%)	30 (71.4%)
	immunosuppression	19 (30.6%)	10 (23.8%)
	not movable to ground tissue	4 (6.4%)	18 (42.9%)
Histopathological high-risk factors			
	infiltration depth above 6 mm	21 (33.9%)	16 (38.1%)
	desmoplasia	0	0
	perineural invasion	17 (27.4%)	11 (26.2%)
	infiltration beyond the subcutis	24 (38.7%)	26 (61.9%)
	poor differentiation	22 (35.5%)	19 (45.2%)
Median tumor diameter in cm (min, max)		3 (1–6.5)	5 (1–18)
Median infiltration depth in mm (min, max)		7 (0.4–24)	6 (1–19)
Comorbidities			
	infection or inflammation	8 (12.9%)	12 (28.5%)
	autoimmune disease	14 (22.6%)	7 (16.6%)
	cardiovascular disease	45 (72.6%)	29 (69.0%)
	immunosuppression	20 (32.2%)	8 (18.0%)
	hematological neoplasm	7 (11.3%)	5 (11.9%)
ECOG status			
	0	33 (53.2%)	13 (31.0%)
	1	22 (35.5%)	24 (57.1%)
	2	5 (8.1%)	3 (7.1%)
	3	2 (3.2%)	2 (4.8%)
Number of blood samples analyzed for CFH			
	Pre-treatment	62	42
	Week 6	NA	19
	Week 12	NA	19
Mean CFH levels in pg/mL (min, max)			
	Pre-treatment	367 (122–895)	465 (172–1307)
	Week 6	NA	319 (252–511)
	Week 12	NA	343 (215–484)
Median follow-up (months)		NA	10

**Table 2:** Univariate and multivariate logistic regression analysis of hi-cSCC and adv-cSCC groups. Logistic regression analysis for parameters discriminates high-risk cutaneous squamous cell carcinoma (hi-cSCC) from advanced cSCC (adv-cSCC). OR: odds ratio, CI: confidence interval, ECOG: Eastern Cooperative Oncology Group; CFH: complement factor H.

	Univariable		Multivariable	
	OR (CI)	*p*-Value	OR (CI)	*p*-Value
Pre-treatment CFH (low)	**0.17 (0.02–0.73)**	**0.030**	**0.13 (0.02–0.7)**	**0.026**
Number of clinical high-risk factors (high)	**2 (1.29–3.23)**	**0.002**	**2.18 (1.3–3.83)**	**0.004**
Number of total high-risk factors (high)	**1.43 (1.12–1.86)**	**0.005**	1.23 (0.85–1.81)	0.274
ECOG (1)	**2.77 (1.18–6.72)**	**0.020**	2.61 (0.96–7.43)	0.063
Inflammatory condition	0.29 (0.06–0.98)	0.066		
Immunosuppression status	0.49 (0.18–1.23)	0.14		
Amount of histological high-risk factors (high)	1.26 (0.92–1.75)	0.150		
Tumor diameter (high)	1.1 (0.96–1.29)	0.184		
Autoimmune disease	0.69 (0.24–1.83)	0.462		
Sex (female)	0.78 (0.3–1.96)	0.609		
Cardiovascular disease	0.84 (0.36–2.01)	0.697		
Infiltration depth (high)	0.99 (0.89–1.08)	0.768		
Age at diagnosis (high)	0.99 (0.95–1.04)	0.819		
Hematological neoplasm	1.06 (0.29–3.58)	0.923		

**Table 3:** Univariate and multivariate Cox proportional hazards analysis in adv-cSCC. Cox proportional hazards analysis of parameters in predicting progress or no progress upon cemiplimab treatment in advanced cutaneous squamous cell carcinoma (adv-cSCC). HR: hazards ratio, CI: confidence interval, ECOG: Eastern Cooperative Oncology Group; CFH: complement factor H.

	Univariable		Multivariable	
	HR (CI)	*p*-Value	HR (CI)	*p*-Value
Baseline CFH (low)	**0.36 (0.14–0.94)**	**0.036**	**0.29 (0.1–0.78)**	**0.014**
Immunosuppression status	**3.04 (1.16–7.98)**	**0.024**	**3.8 (1.38–10.42)**	**0.009**
CFH 6 weeks (low)	1 (0.99–1.01)	0.064		
Amount of histological high-risk factors (high)	1.36 (0.94–1.98)	0.103		
Hematological neoplasm	2.42 (0.8–7.32)	0.117		
Amount of total high-risk factors (high)	1.2 (0.94–1.53)	0.140		
Sex (female)	0.44 (0.12–1.6)	0.214		
CFH 12 weeks (low)	1 (0.99–1)	0.319		
ECOG (1)	1.71 (0.55–5.27)	0.353		
Inflammatory condition	2 (0.46–8.77)	0.358		
Tumor diameter (high)	1.04 (0.92–1.18)	0.502		
Amount of clinical high-risk factors (high)	1.11 (0.74–1.67)	0.598		
Infiltration depth (high)	1.04 (0.89–1.21)	0.610		
Autoimmune disease	1.28 (0.43–3.84)	0.657		
Age at diagnosis (high)	1.01 (0.97–1.06)	0.661		
Cardiovascular disease	0.9 (0.36–2.25)	0.822		

## Data Availability

The datasets generated and analyzed during the current study are not publicly available due to privacy regulations and consent restrictions, but are available from the corresponding author upon reasonable request. All data shared will be de-identified in accordance with ethical guidelines.

## Abbreviations

The following abbreviations are used in this manuscript:

cSCC	cutaneous squamous cell carcinoma
ICI	immune checkpoint inhibition
CFH	complement factor H
